# Lattice Deformation of Tb_0.29_Dy_0.71_Fe_1.95_ Alloy during Magnetization

**DOI:** 10.3390/mi14101861

**Published:** 2023-09-28

**Authors:** Jiaxin Gong, Jiheng Li, Xiaoqian Bao, Xuexu Gao

**Affiliations:** State Key Laboratory for Advanced Metals and Materials, University of Science and Technology Beijing, 30 Xue Yuan Road, Beijing 100083, China; gongjiaxin416@163.com (J.G.); lijh@ustb.edu.cn (J.L.); bxq118@ustb.edu.cn (X.B.)

**Keywords:** magnetic materials, Tb_0.29_Dy_0.71_Fe_1.95_ alloy, crystal structure, lattice parameter, Rietveld refinement

## Abstract

In Tb-Dy-Fe alloy systems, Tb_0.29_Dy_0.71_Fe_1.95_ alloy shows giant magnetostrictive properties under low magnetic fields, thus having great potential for transducer and sensor applications. In this work, the lattice parameters of Tb_0.29_Dy_0.71_Fe_1.95_ compounds as a function of a magnetic field were investigated using in situ X-ray diffraction under an applied magnetic field. The results showed that the c-axis elongation of the rhombohedral unit cell was the dominant contributor to magnetostriction at a low magnetic field (0–500 Oe). As the magnetic field intensity increased from 500 Oe to 1500 Oe, although the magnetostrictive coefficient continued to increase, the lattice constant did not change, which indicated that the elongated c-axis of the rhombohedral unit cell rotated in the direction of the magnetic field. This rotation mainly contributed to the magnetostriction phenomenon at magnetic fields of above 500 Oe. The structural origin of the magnetostriction performance of these materials was attributed to the increase in rhombohedral lattice parameters and the rotation of the extension axis of the rhombohedral lattice.

## 1. Introduction

Tb-Dy-Fe alloy is known as giant magnetostrictive material because of its strong magnetostrictive properties, which can be used in transducers, sensors, actuators, and other devices [1,2]. In Tb_l−x_Dy_x_Fe_2_ alloy systems, the composition x = 0.67–0.73 is frequently used and is also the composition used as a giant magnetostrictive material. For a long time, it was thought that Tb_l−x_Dy_x_Fe_2_ had a cubic Laves phase (C15) structure with a lattice parameter of 0.73 nm [3,4]. Its cubic Laves phase compounds, ReFe_2_ (Re = rare earth), are well known to exhibit giant magnetostriction at room temperature [5]. In the compound ReFe_2_, the rare earth spins are taken to be parallel to one another and antiparallel to the iron spins, showing large magnetic anisotropy. Rare earth compounds with iron in the Laves (C15) phase are strongly magnetic well above room temperature [6]. In the C15 crystal structure, each transition metal atom is surrounded by six other atoms as its nearest neighbors. It also had long been believed that Tb_0.3_Dy_0.7_Fe_2_ alloy had a C15-type cubic Laves phase structure across each transition [7]. With the improvement in device resolution, researchers have gained a new understanding of crystal structure. In recent years, synchrotron data have shown that the ferromagnetic transition in ReFe_2_ compounds results in a low crystallographic symmetry conforming to the spontaneous magnetization direction [8,9]. Ferromagnetic Tb_l−x_Dy_x_Fe_2_ materials have been shown to consist of coexisting rhombohedral and tetragonal crystallographic structures at room temperature, as measured via high-resolution X-ray diffraction and AC magnetic susceptibility measurements [8,9,10]. Tb_0.3_Dy_0.7_Fe_2_, a typical composition of the Terfenol-D giant magnetostrictive material (GMM), has been shown to consist of coexisting rhombohedral and tetragonal phases over a wide temperature range, and the local rhombohedral and tetragonal domains can easily respond to a low external magnetic field, thus facilitating easy magnetization rotation and high magnetostrictive properties [11,12,13]. As the resolution of the synchrotron XRD instrument is unable to distinguish small tetragonal distortions from a cubic structure, the tetragonal structure is generally fitted and calculated as a cubic structure [10,14]. The rhombohedral lattice constant of Tb_0.3_Dy_0.7_Fe_2_ was determined by Yang et al. [8] using high-resolution synchrotron radiation XRD equipment as a = 7.336 Å, α = 89.91°. Gong et al. [14] measured the lattice constants of cubic (tetragonal) and rhombohedral structures as a = 7.329 Å and a = 7.334 Å, respectively. After heat treatment, the lattice constant of the sample was deformed by about 1‰, but the magnetostrictive performance of the sample was significantly improved. It was found that although the difference of lattice constants between the two structures is small, the magnetostrictive properties of the two structures change greatly when the crystal structure parameters change slightly. The crystal structure seems to profoundly influence magnetostriction phenomena. Therefore, the structure of Tb-Dy-Fe alloy was used as a standard C15 structure for many years due to the insufficient resolution of the equipment. The relationship between the subtle changes in lattice parameters and magnetostrictive properties needs to be further studied.

The magnetostriction effect is a physical phenomenon in which the shape and size of a material change when it is magnetized. The magnetostriction phenomena of Tb_l−x_Dy_x_Fe_2_ have been fully studied and explained in terms of magnetic domains, the anisotropic energy of magnetic crystals, and domain structures [15,16,17,18]. According to the theory of magnetic domain [19,20,21,22], when a Tb-Dy-Fe material is at a temperature lower than the Curie temperature, it spontaneously magnetizes, forming magnetic domains in various directions. During magnetization, magnetic domain rotation and domain wall displacement occur, resulting in magnetostriction. Nevertheless, magnet domain theory is only a phenomenological theory, which does not involve any crystal structure parameters, only describing macroscopic phenomena. The theory of magnetostriction still needs to be studied and improved. In order to obtain magnetostrictive materials with higher performance, we generally need to regulate the materials. At present, Tb-Dy-Fe alloy is generally regulated via heat treatment. Most of the heat treatment methods used involve improving the magnetostrictive properties of Tb-Dy-Fe materials with uniform composition, uniform phase structure, and specific magnetic domain orientation [23,24,25]. However, there is no perfect theory guiding the regulation of the Tb-Dy-Fe crystal structure. The deformation of the crystal structure could be another factor that greatly influences magnetostrictive performance. For Tb_l−x_Dy_x_Fe_2_ compounds, one of their prominent features is their localized 4f electrons and itinerant 3d electrons, and the 4f electrons of Tb and Dy make the main contribution to magnetostriction [17]. When the sample is magnetized by an external magnetic field, the distribution of 4f electrons related to the crystal electric field also changes accordingly. Changes in size or orientation of the Tb and Dy magnetic moment are reflected in a change in the 4f charge distribution, which in turn forces the surrounding atoms to attain new equilibrium positions, minimizing the total energy [6]. That is to say, this series of changes produces lattice deformation in the crystal structure, and the end result is the phenomenon of magnetostriction. As a consequence, large magnetostriction originates from magnetic-field-induced large lattice deformation [26]. However, due to the lack of measurement accuracy, the fact that the texture in a directionally solidified sample is too strong to obtain an accurate lattice constant, and the fact that a powder sample easily moves in a magnetic field, measurement of the lattice deformation of Tb-Dy-Fe under different magnetic field distributions remains a challenge. There have been few studies on crystallography during magnetization, and the lattice deformation resulting from magnetostriction at low fields is poorly understood. Therefore, it is necessary to conduct some further research on the lattice deformation of Tb-Dy-Fe alloy during magnetization.

In the present study, the crystal structure and lattice deformation of polycrystalline compounds with the nominal composition Tb_0.29_Dy_0.71_Fe_1.95_ during magnetization were investigated. We aimed to understand the deformation of the crystal structure during magnetostriction and to gain a deeper understanding of magnetostriction. We also hoped to provide theoretical guidance for improving magnetostrictive properties by regulating crystal structure.

## 2. Material and Methods

**Figure 1:**
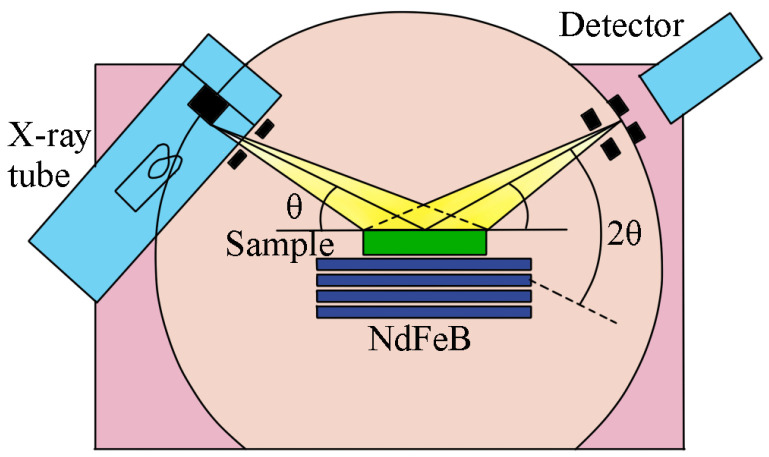
Schematic of the experimental setup for XRD. By stacking NdFeB magnets, magnetic fields of up to 2500 Oe were generated. The direction of the magnetic inductance lines is perpendicular to the X-ray scan surface.

In order to obtain excellent magnetostrictive properties from Tb-Dy-Fe alloys, it is of crucial importance to fabricate oriented polycrystalline crystal via directional solidification. An alloy with the nominal composition Tb_0.29_Dy_0.71_Fe_1.95_ was prepared from highly pure Fe (99.9 wt.%), Tb (99.99 wt.%), and Dy(99.99% wt.%) via the Bridgeman directional solidification process in an argon atmosphere. Then, the alloy was annealed at 1060 °C for 2 h in an argon atmosphere. This composition ratio of the alloy ensured that the main phase was all RFe_2_ phase without any RFe_3_ phase, as shown in reference [14]. The directionally solidified alloys prepared via this common method have a strong texture, with <110> axial preferred orientation generally. Thus, the diffraction peaks of many other crystal planes are very low or do not appear in the XRD patterns. To characterize the general law of magnetostriction, an isotropic sample was prepared by grinding the directionally solidified samples into a 25~40 µm powder mixed with a small amount (about 5 wt.%) of epoxy resin, so that the powder would not move or freely rotate in an external magnetic field. The powder was ground by hand instead of using a high-energy ball mill. The particle size of the powder was screened using a standard sieve. It should be noted that these operations were all carried out in an argon atmosphere glove box. The powder was not exposed to the air, as much as possible, to avoid oxidation. The isotropic sample was cured in an argon atmosphere for more than 24 h; after the end of curing, the sample would not easily oxidize. X-ray powder diffraction (XRD) patterns were obtained with Cu-Kα radiation (with wavelengths λ-Kα_1_ = 1.54059 Å and λ-Kα_2_ = 1.54431 Å) on a Rigaku (Smart Lab 9Kw, Tokyo, Japan) X-ray diffractometer, and the step scan increment (2θ) was 0.004 degrees. The sample table of the X-ray diffractometer was improved by using nonmagnetic material, and an in situ magnetic field experiment was carried out. By using a fixed device, the sample remained in a fixed position. In the process of changing the number of NdFeB magnets, the position of the sample remained unchanged. The magnetic field intensity was controlled during the in situ magnetic field XRD process by increasing the number of NdFeB magnets, as shown in Figure 1. The magnetic field on the upper surface of the sample was monitored using a Hall probe. All the obtained patterns were analyzed using the Rietveld method and Fullprof software (https://www.ill.eu/sites/fullprof/php/downloads.html, accessed on 29 November 2022). A measuring device for magnetostrictive materials using a strain gauge was employed to measure the magnetostriction coefficient of this powder-bonded sample.

## 3. Results and Discussion

**Figure 2:**
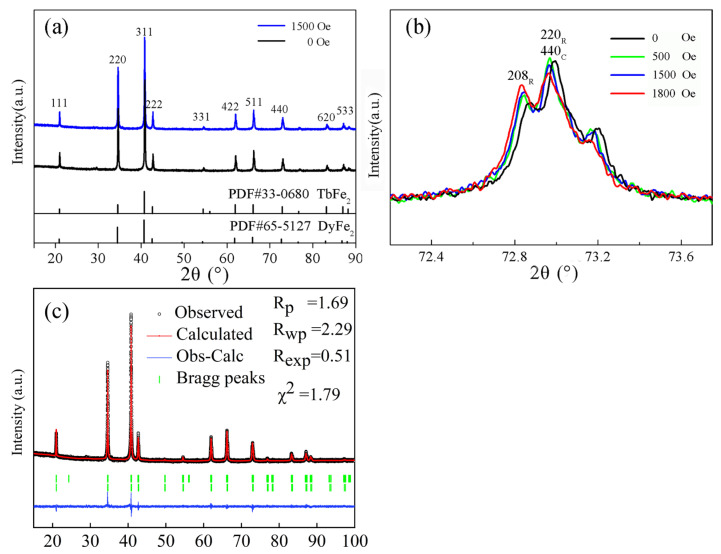
XRD patterns of the Tb_0.29_Dy_0.71_Fe_1.95_ sample under different magnetic fields. (**a**) XRD patterns at magnetic fields of 0 and 1500 Oe using a cubic structure index, compared with standard PDF cards. (**b**) The peak shape at 2θ = 73° formed by the superposition of rhomboidal and cubic structure peaks, and the intensity of the small peak on the right is half of the (220_R_ + 440_C_) peak, indicating the Ka_2_ diffraction peak, which had no influence on the analysis. (**c**) Plot of the Rietveld refinement of the XRD diffraction pattern recorded at 1500 Oe. The first and second rows of green Bragg peaks refer to the hexagonal and cubic types of Tb-Dy-Fe, respectively.

Figure 1 shows a schematic diagram of the in situ magnetic field XRD experiment. A cylindrical sample with a thickness of 4.5 mm was fixed on the sample rack, and several NdFeB magnets with a thickness of 2 mm were applied below. Based on measurements of the magnetic field on the upper surface of the sample with a Hall sensor, magnetic fields of 250 Oe, 500 Oe, 850 Oe, 1200 Oe, 1500 Oe, 1800 Oe, and 2500 Oe were obtained. Magnetostriction mainly occurred in the axial direction of the sample, and the plane scanned by the X-rays was perpendicular to the direction of the magnetostriction. Figure 2a shows the XRD patterns of the sample at room temperature under different magnetic fields. Comparing the XRD pattern of the 0 magnetic field with the standard PDF cards #33-0680 and #65-5127, the positions, quantity, and the relative strength of the diffraction peaks were all similar to the standard sample. The patterns indicated that the sample was isotropic without texture, with a typical ReFe_2_ (Re = Tb, Dy) Laves phase. The sample was tested in different magnetic fields ranging from 0 to 2500 Oe. After the application of a magnetic field of 1500 Oe, there was still no obvious change in the peak relative strengths, indicating no obvious texture. However, a closer comparison of the 440 peaks revealed subtle changes in the position and intensity of the peaks, as shown in Figure 2b. This indicated a change in the lattice constants or crystal orientation. The peak pattern consisted of cubic 440 and rhombic 208, 220 peaks, which are the same as those reported in the literature, indicating the coexistence of a cubic structure and a rhombohedral structure in the crystal [27,28]. To obtain accurate lattice parameters, the Rietveld refinement [29,30,31] method was used to fit the full XRD patterns. The XRD patterns of eight different magnetic fields were refined in the same process. For example, the fitting of the full XRD pattern obtained under a 1500 Oe magnetic field is shown in Figure 2c.

**Figure 3:**
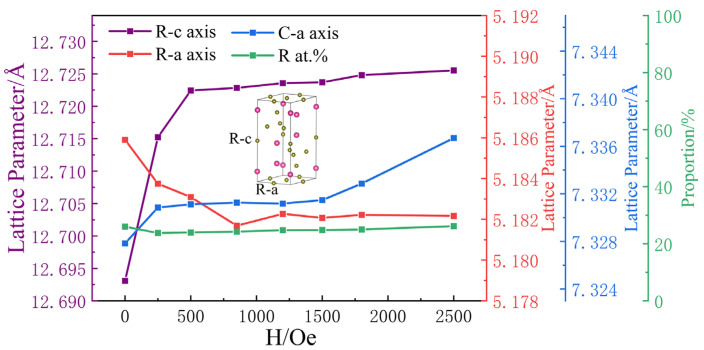
Magnetic field dependence of the lattice parameters and rhombohedral structure proportions of Tb_0.29_Dy_0.71_Fe_1.95_. The illustration shows a diagram of the rhombohedral R$\bar{3}$m(H) model (No. 166). R-c and R-a are the c-axis and a-axis lattice parameters of the rhombohedral structure, respectively. C-a is the lattice parameter of the cubic structure.

The tetragonal structure was fitted with the cubic Fd$\bar{3}$m symmetry [6,7], as the distortion of the tetragonal structure was too small to be distinguished from the cubic structure using XRD [4,6]. The rhombohedral R$\bar{3}$m(H) (No. 166) model and Fd$\bar{3}$m (No. 227) model were adopted for the fitting, as in the literature [6,17]. The space group R$\bar{3}$m (No. 166) characterizes the rhombohedral crystal structure, which can be equivalently described by the hexagonal crystal structure R$\bar{3}$m(H). The hexagonal (rhombohedral) crystal structure of Tb-Dy-Fe is equal to a distortion of the Laves cubic structure along the [111] direction [17]. The [0001] direction (c-axis) of the hexagonal structure is parallel to the [111] direction of the cubic structure, while the [10$\bar{1}$0] direction (a-axis) of the hexagonal structure is parallel to the [110] direction of the cubic structure. In the R$\bar{3}$m(H) crystal structure model, a = b ≠ c, α = β = 90° and γ = 120°; the Tb and Dy atomic position coordinates are both (0, 0, 0.125); Fe atoms exist in two positions, (0, 0, 0.5) and (0.5, 0, 0). In the Fd$\bar{3}$m model, a = b = c, α = β = γ = 90°; the Tb and Dy atomic position coordinates are both (0, 0, 0); and the Fe atomic coordinate is (0.625, 0.625, 0.625). During refinement, we mainly refined the lattice parameters, scale factors, preferred orientation, asymmetry parameters, shape parameters, and global parameters such as instrumental profile, background, and so on. As the characteristics of the Tb_1−x_Dy_x_Fe_2_ crystal, as Tb and Dy atoms are similar in size, as their characteristic peaks are difficult to distinguish accurately in X-ray diffraction, and considering the characteristics of the R$\bar{3}$m(H) (No. 166) and Fd$\bar{3}$m cell models, the site occupancy (Occ) and isotropic thermal parameter (B) were not used as the focus of refinement. The results of the refinement procedure with satisfactory fits, including lattice parameters, cell volume, and phase fraction of the Tb_0.29_Dy_0.71_Fe_1.95_ compound in the magnetization state, are presented in Table 1. The satisfactory fit parameters of all full-pattern fittings are small (χ^2^ < 2) and within a reasonable range. The displacement errors of the instrument during the refinement were equal for all XRD patterns, so the final results accurately indicated the relative changes in the crystal structure parameters. In order to show the lattice parameters intuitively, we drew Figure 3 with the main parameters.

Figure 3 shows the variation in the crystal structure parameters with the magnetic field obtained through refinement. The c-axis lattice parameter of the rhombohedral structure (R-c) was equivalent to the cubic structure lattice parameter expanded along the <111> direction, that is, the easy magnetization axis direction. Between 0 and 500 Oe, the most obvious change was in the c-axis lattice parameter of the rhombohedral structure, with an elongation of approximately 2.4 parts per thousand. In addition, the a-axis lattice parameter of the rhombohedral structure (R-a) decreases. The rate of change in the cell volume was calculated to be between 0.2 and 0.65 parts per thousand (shown in Table 1), which is an order of magnitude smaller than the rate of change in R-c. We noted that the lattice constant of the cubic structure (C-a) increased slightly at 250 Oe and then flattened out, until it exceeded 1500 Oe. The reason may be that a magnetic field of 0–250 Oe in size can overcome a low energy barrier and increase C-a slightly. If C-a continued to increase, a larger magnetic field was needed to overcome the high energy barrier. The rate of change in the lattice parameter of the cubic structure was approximately 0.4 parts per thousand. Therefore, the R-c elongation of the rhombohedral crystal mainly contributed to the magnetostriction phenomenon under a low magnetic field.

**Figure 4:**
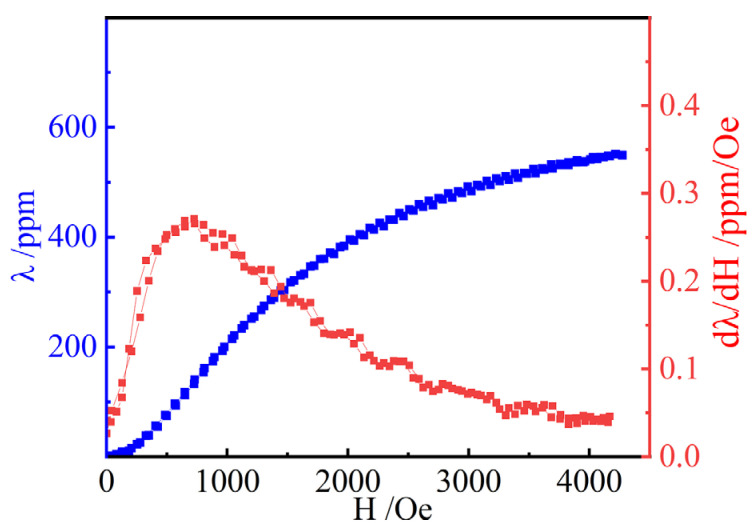
Intrinsic magnetostriction curves and dλ/dH curves of bonded Tb_0.29_Dy_0.71_Fe_1.95_ under free compressive stress.

**Figure 5:**
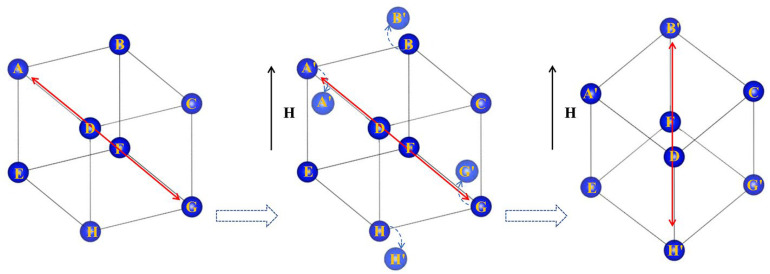
The extension direction of the lattice rotates towards the magnetic field direction. H represents the applied magnetic field. The letters A–G represent the location of the atom.

The lattice parameters of both the rhombohedral and cubic structures did not significantly change between magnetic field intensities of approximately 500 Oe and 1500 Oe. However, the magnetostriction coefficient of the powder-bonded sample still increased with increasing magnetic field intensity, as shown by the blue curve in Figure 4. Therefore, we propose that the orientation of the rhombohedral crystal structure is arranged in different directions and can rotate under the action of a magnetic field. This view can be confirmed in Figure 2c, where the relative strength of rhombohedral peak 208_R_ and (220_R_ + 440_C_) peaks varies in a magnetic field with a ratio of 0.73 at 500 Oe and 0.80 at 1500 Oe. Since the results of the Rietveld refinement show that the ratio of rhomboidal structure to cubic structure remains unchanged, that is, the relative strength of the 440c peaks does not change, the relative strength of the 208_R_ and 220_R_ peaks should change in the magnetic field, which means that the Rhomboidal structure is oriented in the magnetic field. However, the crystal cell does not actually rotate, according to the principle of energy minimization [6,12]; but, through the displacement of atoms to a nearby position by overcoming the lowest barrier, the direction of extension of the crystal lattice is rotated, as shown in Figure 5. The rhombohedral c-axis elongation in the sample may be along any direction in the initial phase. When the direction is inconsistent with the magnetic field H, with increasing magnetic field, the atoms overcome the barrier and move towards a nearby position. In Figure 3, the atoms in positions A, G, B, and H move towards positions A′, G′, B′, and H′, respectively. After all the atoms (including atoms in the C, D, E, and F positions) have moved to new equilibrium positions, the elongation direction of the cell is rotated from the initial AG direction to the H′B′ direction, which is parallel to the magnetic field. The rhombohedral cell “rotation”, in this way, mainly contributes to the magnetostriction phenomenon for 500–1500 Oe magnetic fields.

Similar to how a magnetic domain is deflected in the direction of the magnetic field [32], the lattice is also deflected in the direction of the magnetic field. We found that this process is reversible and repeatable through the process of magnetostrictive coefficient measurement. Therefore, we think that the increase in the magnetostriction coefficient in the magnetic field at 500 Oe–1500 Oe is due to the gradual rotation of the R-c direction of the rhombohedral lattice; the R-c whose initial direction is not in line with the magnetic field direction gradually shift to the magnetic field direction. This rotation process may continue until the magnetic field exceeds 2500 Oe and approaches the saturation magnetic field. The linear magnetostriction for high magnetic fields is mainly caused by rotation, and the rate of magnetostriction gradually decreases with the increase in the magnetic field. Since the main change occurring after the magnetic field intensity exceeds 2000 Oe is the growth in the cubic lattice parameter C-a, and it is thought that 2000 Oe can overcome the barrier of continued expansion of the cubic lattice, volume magnetostriction [33] may begin at this point. We note that the proportions of the rhombohedral structure and cubic structure hardly change, so the change in lattice parameters is the main factor in this magnetization process.

Dynamic magnetostriction (d_33_) was calculated using dλ/dH, as shown by the red curve in Figure 4. The higher the value of dλ/dH, the lower the field needed to trigger large magnetostriction, and the more sensitive the sample deformation to the magnetic field. In practical applications, the Tb-Dy-Fe alloys with a higher dλ/dH can help realize the miniaturization of devices. The largest dλ/dH appears at about 700 Oe and reaches the maximum value of 0.3 ppm/Oe. In the range of 500 Oe–900 Oe, dλ/dH maintains a relatively large value. When the rhomboidal structure begins to rotate, the sample deformation is most sensitive to changes in the magnetic field. It can be deduced that the highest contribution efficiency of rhomboidal lattice deformation to magnetostriction occurs after the beginning of rotation. When the magnetic field exceeds 700 Oe, the dλ/dH value slowly decreases from the highest value. This is because rhombohedral structures with certain favorable angles to the direction of the magnetic field rotate preferentially, and rhombohedral structures with other angles rotate successively after the magnetic field continues to increase. This results in a departure from the linearity of the response of the lattice strain to the applied magnetic field and easily increase the saturation magnetic field. If there are regulatory measures to make the R-c direction of all rhomboid cells rotate at the most favorable rotation position in the beginning and rotate together after reaching 500 Oe, the saturation magnetic field greatly reduces and the dλ/dH increases. This topic clearly needs further study.

The magnetostrictive curve of Tb-Dy-Fe has complex nonlinearity, which seriously limits the accuracy of device control. The more linear the performance curve of a magnetostrictive material, the more accurate the microdevices made of it in application [34]. This requires dλ/dH to decline as slowly as possible after reaching the highest value. From the perspective of crystal structure, the more rhomboidal structures rotate and the longer the distance of rotation, the better the linearity of the magnetostrictive curve. In other words, via crystal structure regulation, the initial direction of R-c elongation of more rhomboidal structures is perpendicular to the magnetic field direction, which improves the linearity of the magnetostrictive curve. Considering symmetry, the longest rotation path occurs when the initial direction is 90 degrees from the final direction. These results give us a deep understanding of the crystal structure of Tb-Dy-Fe and the magnetostriction principle in Tb-Dy-Fe materials from the perspective of crystal structure deformation.

## 4. Conclusions

In conclusion, we performed XRD studies on Tb_0.29_Dy_0.71_Fe_1.95_ compounds under different magnetic fields and employed the Rietveld method to refine the XRD patterns. Rhomboidal cells play an important role in linear magnetostriction. The elongation of the rhombohedral structure along the c-axis under a low magnetic field (0–500 Oe) was evidenced. The rhombohedral crystal structures were randomly oriented in the case without a magnetic field, and the application of a magnetic field yielded rhombohedral crystal structure rotation. The model of crystal structure rotation was given. The c-axis direction of the R$\bar{3}$m(H) symmetrical crystal structure was arranged in every direction, and rearrangement along the magnetic field direction mainly occurred after the magnetic field strength exceeded 500 Oe. Shortly after the rhomboidal structure began to rotate (under a 700 Oe magnetic field), the resulting strain was most sensitive to changes in the magnetic field, and dλ/dH reached its maximum value. Conversion between the rhombohedral and cubic structures was rare under the magnetic fields. Therefore, the main source of magnetostriction was not the transformation of the crystal structure but the change in the lattice parameters and the rotation of the extension axis of the rhombohedral lattice.

## Figures and Tables

**Table 1:** Lattice parameters, cell volume, phase fraction, and satisfactory fits of XRD patterns of Tb_0.29_Dy_0.71_Fe_1.95_ compound in the magnetization state. To facilitate the distinction between lattice constants of different structures, R-a, R-c, and C-a are defined.

Magnetic Field (Oe)	$R\bar{3}$m(H)			$\mathrm{Fd}\bar{3}$m		Fraction (wt.%)	χ^2^
	a = b(R-a) (Å)	c(R-c) (Å)	V (Å^3^)	a = b = c(C-a) (Å)	V (Å^3^)		
0	5.1866(1)	12.6903(5)	295.64(2)	7.3275(1)	393.43(1)	25.9(2)	1.69
250	5.1838(2)	12.7152(1)	295.90(3)	7.3309(1)	393.97(1)	23.8(2)	1.72
500	5.1831(2)	12.7224(1)	296.09(2)	7.3311(1)	394.01(1)	24.0(2)	1.68
850	5.1817(1)	12.7228(2)	295.76(1)	7.3313(1)	394.03(1)	24.3(2)	1.70
1200	5.1822(1)	12.7236(3)	295.98(1)	7.3311(1)	394.00(1)	24.8(1)	1.82
1500	5.1820(1)	12.7237(4)	295.88(1)	7.3315(1)	394.07(1)	24.8(2)	1.79
1800	5.1822(1)	12.7248(1)	295.95(1)	7.3329(1)	394.29(1)	25.0(2)	1.71
2500	5.1821(1)	12.7256(1)	295.94(1)	7.3367(1)	394.91(1)	26.2(3)	1.76

## Data Availability

Data is contained within the article. The data presented in this study are available in this article.

## References

[1] Claeyssen F., Lhermet N., Le Letty R., Bouchilloux P. (1997). Actuators, transducers and motors based on giant magnetostrictive materials. J. Alloys Compd..

[2] Jammalamadaka S.N., Markandeyulu G., Kannan E., Balasubramaniam K. (2008). Development of a magetnetostrictive transducer for nondestructive testing of concrete structures. Appl. Phys. Lett..

[3] Palit M., Banumathy S., Singh A.K., Pandian S. (2011). Crystallography of solideliquid interface and evolution of texture during directional solidification of Tb_0.3_Dy_0.7_Fe_1.95_ alloy. Intermetallics.

[4] Clark A.E., Cullen J.E., McMaster O.D., Callen E.B. (1976). Rhombohedral distortion in highly magnetostrictive Laves phase compounds. Am. Inst. Phys. Conf. Proc..

[5] Clark A.E. (1979). Chapter 15 Magnetostrictive RFe_2_ intermetallic compounds. Handbook on the Physics and Chemistry of Rare Earths.

[6] Cullen J.R., Clark A.E. (1977). Magnetostriction and structural distortion in rare-earth intermetallics. Phys. Rev. B.

[7] Atzmony U., Dariel M.P., Dublon G. (1977). Spin-orientation diagram of the pseudobinary Tb_1−x_Dy_x_Fe_2_ Laves compounds. Phys. Rev. B.

[8] Yang S., Ren X.B. (2008). Noncubic crystallographic symmetry of a cubic ferromagnet: Simultaneous structural change at the ferromagnetic transition. Phys. Rev. B.

[9] Yang S., Bao H.X., Zhou C. (2010). Large Magnetostriction from Morphotropic Phase Boundary in Ferromagnets. Phys. Rev. Lett..

[10] Richard B., Manfred W., James C. (2013). Morphotropic Phase Boundaries in Ferromagnets: Tb_1−x_Dy_x_Fe_2_ Alloys. Phys. Rev. Lett..

[11] Ma T.Y., Liu X.L., Pan X.W. (2014). Local rhombohedral symmetry in Tb_0.3_Dy_0.7_Fe_2_ near the morphotropic phase boundary. Appl. Phys. Lett..

[12] Ma T.Y., Zhang C.S., Zhang J.J. (2010). Magnetic force microscopy study of magnetically annealed Tb_0.36_Dy_0.64_(Fe_0.85_Co_0.15_)_2_ polycrystals. Appl. Phys..

[13] Zhang C.S., Sun G.G., Mi M.Y., Ma T.Y. (2014). Effect of the induced anisotropy axis on altering domain alignment and magnetostriction of Terfenol-D. Appl. Phys. Lett..

[14] Gong J.X., Li J.H., Bao X.Q., Hou R.F., Gao X.X. (2022). A study of the crystal structure of a Tb-Dy-Fe alloy during annealing via rietveld analysis. Intermetallics.

[15] Jiboory A., Lord D.G. (1993). Magnetic domains and microstructural defects in Terfenol-D. J. Appl. Phys..

[16] Jiles D.C., Thoelke J.B. (1994). Theoretical modelling of the effects of anisotropy and stress on the magnetization and magnetostriction of Tb_0.3_Dy_0.7_Fe_2_. J. Magn. Magn. Mater..

[17] Hu C.C., Shi Y.G., Shi D.N. (2013). Anisotropy compensation and magnetostrictive properties in Tb_x_Dy_1-x_(Fe_0.9_Mn_0.1_)_1.93_ Laves compounds: Experimental and theoretical analysis. J. Appl. Phys..

[18] Zhang C.S., Ma T.Y., Pan X.W., Mi Y. (2012). Domain Rotation Simulation of the Magnetostriction Jump Effect of (110) Oriented TbDyFe Crystals. Chin. Phys. Lett..

[19] Armstrong W.D. (2003). An incremental theory of magneto-elastic hysteresis in pseudo-cubic ferro-magnetostrictive alloys. J. Magn. Magn. Mater..

[20] Armstrong W.D. (2003). Fully three-dimensional incremental model of magneto-elastic hysteresis in Terfenol-D. Smart Mater. Struct..

[21] Jiles D.C., Thoelke J.B. (1991). Modeling of the combined effects of stress and anisotropy on the magnetostriction of Tb_0.3_Dy_0.7_Fe_2_. IEEE Trans. Magn..

[22] Mei W., Okane T., Umeda T. (1998). Magnetostriction of Tb–Dy–Fe crystals. J. Appl. Phys..

[23] Verhoeven J.D., Ostenson J.E., Gibson E.D., McMasters O.D. (1989). The effect of composition and magnetic heat treatment on the magnetostriction of Tb_x_Dy_1−x_Fe_y_ twinned single crystals. J. Appl. Phys..

[24] Prajapati K., Greenough R.D., Jenner A.G. (1994). Device oriented magnetoelastic properties of Tb_x_Dy_1−x_Fe_1.95_ (x = 0.27, 0.3) at elevated temperatures. J. Appl. Phys..

[25] Wu W., Zhang M.C., Gao X.X., Zhou S.Z. (2006). Effect of two-steps heat treatment on the mechanical properties and magnetostriction of <1 1 0> oriented TbDyFe giant magnetostrictive material. J. Appl. Phys..

[26] Hui Z., Ji Y.C., Ma T.Y., Yang S., Ren X.B. (2021). Exceptional combination of large magnetostriction, low hysteresis and wide working temperature range in Tb_1−x_Fe_2−x_DyCo_2_ alloys. Acta Mater..

[27] Sayetat F. (1975). X-ray powder diffraction at low temperature applied to the determination of magnetoelastic properties in terbium iron garnet. J. Appl. Phys..

[28] Dwight A.E., Kimball C.W. (1974). TbFe_2_, A rhombohedral Laves phase. Acta Crystallogr. B.

[29] Roisnel T., Rodríquez-Carvajal J. (2001). WinPLOTR: A Windows Tool for Powder Diffraction Pattern Analysis. Epdic 7: European Powder Diffraction. MSF.

[30] Rodriguez-Carvajal J., Roisnel T. FullProf. 98 and WinPLOTR: New Windows 95/NT Applications for diffraction. Commission for Powder Diffraction, International Union of Crystallography, Newsletter 20. May–August 1998. http://www.ill.eu/sites/fullprof/.

[31] Rodriguez-Carvajal J. (1993). Recent advances in magnetic structure determination by neutron powder diffraction. Phys. B.

[32] Slim N., Sonia B., Christian V., Olfa K., Slim C. (2016). Investigation of the magnetostrictive effect in a Terfenol-D plate under a non-uniform magnetic field by atomic force microscopy. Mater. Des..

[33] Teter J.P., Clark A.E., Wun-Fogle M. Large rotation induced volume magnetostriction in Tb_0.27_Dy_0.73_Fe_2−x_. Proceedings of the International Magnetics Conference.

[34] Seco F., Martin J.M., Jimdnez A.R. (2005). A high accuracy magnetostrictive linear position sensor. Sens. Actuators A.

